# Thromboembolic and Mortality Risk in Patients with Postoperative Atrial Fibrillation After Coronary Artery Bypass Graft—The TRIP-AF Study

**DOI:** 10.3390/jcm15197577

**Published:** 2026-09-29

**Authors:** Roberto F. E. Pedretti, Lucio Addeo, Pasquale Campana, Fabio Angeli, Maurizio Bussotti, Angelo Caporotondi, Gaia Cattadori, Valentina Cremona, Lia Crotti, Maria De Salvo, Mario Facchini, Anna Frisinghelli, Paola Grati, Niccolò B. Grieco, Maria Teresa La Rovere, Antimo Papa, Andrea Passantino, Martina Passera, Anna Picozzi, Franco Tarro Genta, Vittorio Racca, Vincenzo Rizza, Maria Cristina Rossi, Simona Sarzi Braga, Simonetta Scalvini, Raffaella Vaninetti, Elio Venturini, Emanuela Zanelli, Alfonso Galati, Mario Mallardo, Laura Dalla Vecchia

**Affiliations:** 1 School of Medicine and Surgery, University of Milano Bicocca, 20126 Milan, MI, Italy; 2 Department of Advanced Biomedical Sciences, University of Naples Federico II, 80131 Naples, NA, Italy; 3 Cardiology Department, Pineta Grande Hospital, 81030 Caserta, CE, Italy; 4 Department of Medicine and Technological Innovation, University of Insubria, 21100 Varese, VA, Italy; 5 Department of Cardiology, Istituti Clinici Scientifici Maugeri IRCCS, 21049 Tradate, VA, Italy; 6 Department of Cardiology, Istituti Clinici Scientifici Maugeri IRCCS, 20138 Milan, MI, Italylaura.dallavecchia@icsmaugeri.it (L.D.V.); 7 Department of Cardiology, Istituti Clinici Scientifici Maugeri IRCCS, 27040 Montescano, PV, Italymariateresa.larovere@icsmaugeri.it (M.T.L.R.); 8 Cardiovascular Department, IRCCS MultiMedica, 20123 Milan, MI, Italy; gaia.cattadori@multimedica.it (G.C.); simona.sarzibraga@multimedica.it (S.S.B.); 9 Cardiovascular Section, Department of Clinical Science and Community Health, University of Milan, 20122 Milan, MI, Italy; 10 Cardiac Rehabilitation, Casa di Cura Le Terrazze, 21035 Cunardo, VA, Italy; v.cremona@clinicaleterrazze.com (V.C.); m.passera@clinicaleterrazze.com (M.P.); 11 Rehabilitation Unit, Department of Cardiology, San Luca Hospital, IRCCS, Istituto Auxologico Italiano, 20149 Milan, MI, Italy; 12 Department of Cardiology, Istituti Clinici Scientifici Maugeri IRCCS, 27100 Pavia, PV, Italy; 13 Cardiac Rehabilitation, Specialist Rehabilitation Unit, Hospital of Passirana di Rho, ASST Rhodense, 20017 Passirana di Rho, MI, Italy; 14 Cardiac Rehabilitation Unit, IRCCS Fondazione Don Gnocchi, Onlus, 20148 Milan, MI, Italyvittorio.racca@unimi.it (V.R.); 15 Cardiovascular Rehabilitation and Pneumology, ASST Centro Specialistico Ortopedico Traumatologico Pini-CTO, 20122 Milan, MI, Italy; 16 Department of Cardiology, Istituti Clinici Scientifici Maugeri IRCCS, 82037 Telese Terme, BN, Italy; 17 Department of Cardiology, Istituti Clinici Scientifici Maugeri IRCCS, 70124 Bari, BA, Italy; 18 Department of Cardiology, Istituti Clinici Scientifici Maugeri IRCCS, 28013 Veruno, NO, Italy; franco.tarrogenta@icsmaugeri.it; 19 San Raffaele Scientific Institute, 20132 Milan, MI, Italy; rizza.vincenzo@hsr.it; 20 Department of Cardiology, Istituti Clinici Scientifici Maugeri IRCCS, 25065 Lumezzane, BS, Italy; simonetta.scalvini@icsmaugeri.it (S.S.);; 21 Cardiac Rehabilitation Unit, Civil Hospital, 57023 Cecina, LI, Italy; 22 Cardiac Rehabilitation Unit, Villa Betania Hospital, 00139 Rome, RM, Italy; 23 Cardiac Rehabilitation Unit, ASL Napoli 1 Centro, 80145 Naples, NA, Italy

**Keywords:** atrial fibrillation, postoperative atrial fibrillation, coronary artery bypass, cardiac rehabilitation, stroke, thromboembolism, mortality

## Abstract

**Background/Objectives**: Postoperative atrial fibrillation (POAF) is a common complication of coronary artery bypass grafting (CABG) and may recur during cardiac rehabilitation (CR); its long-term prognostic value remains unclear. We evaluated the impact of POAF on a composite endpoint of all-cause mortality and non-fatal stroke after isolated CABG and assessed POAF duration and timing, AF recurrence, and other predictors of adverse outcomes. **Methods**: This multicentre retrospective study enrolled 1139 patients who underwent CR after isolated CABG between 2011 and 2015 across 17 Italian centres. Clinical and outcome data were obtained from medical records and structured interviews; median follow-up was 5.78 years. **Results**: POAF occurred in 34.3% of patients and was associated with a higher incidence of the primary composite endpoint of all-cause mortality and non-fatal stroke in univariate analysis (15.4% vs. 10.0%, *p* = 0.008), particularly when lasting ≥48 h or occurring during both surgery and rehabilitation. However, in multivariate analysis POAF was not an independent predictor; instead, CHA_2_DS_2_-VASc score ≥3, reduced left ventricular ejection fraction, elevated urea, history of heart failure, and AF recurrence during follow-up predicted adverse outcomes. **Conclusions**: POAF reflects early vulnerability but does not independently predict long-term mortality or stroke. Conversely, AF recurrence and CHA_2_DS_2_-VASc score retain strong prognostic value, underscoring the need for long-term rhythm monitoring and individualised risk assessment post-CABG.

## 1. Introduction

Atrial fibrillation (AF) is the most common arrhythmia following cardiac surgery (CS), with reported rates up to 40% after coronary artery bypass grafting (CABG) and even higher after combined procedures. Postoperative AF (POAF) typically occurs in the early postoperative period but is also frequent during cardiac rehabilitation (CR), affecting up to 17% of patients [1]. The 2024 ESC guidelines acknowledge the association between POAF and increased early stroke risk, morbidity, and 30-day mortality. There is still a lack of data regarding the long-term impact of POAF and the prognostic significance of its duration. This study aimed to evaluate, in a cohort of patients undergoing isolated CABG and early in-hospital CR, the impact of POAF on a composite endpoint of all-cause mortality and non-fatal stroke over a 5-year follow-up. Secondary objectives included the prognostic impact of arrhythmia duration (e.g., ≥48 h), the association between AF recurrence and adverse outcomes and identification of independent predictors for adverse outcomes.

## 2. Materials and Methods

### 2.1. Study Design and Population

TRIP-AF was an observational, multicentre study, which enrolled a retrospective cohort collecting prospective follow-up data. A cohort of 1139 patients who underwent in-hospital CR after isolated CABG in the years 2011–2015 in 17 centres in Italy was enrolled in the study. Data (main clinical and demographic characteristics) were collected retrospectively from patients’ records, including files from CS records. CHA_2_DS_2_-VASc and HAS-BLED scores were calculated for each patient by patients’ records available during the in-hospital CR programmeme. Only patients undergoing isolated CABG were included; concomitant valve or other cardiac surgery was an exclusion criterion as well as in-hospital death before CR admission. The CHA2DS2-VASc and HAS-BLED scores were computed from data available at admission to in-hospital CR and therefore reflect baseline status before any in-hospital follow-up event, avoiding post-event (collider) bias.

### 2.2. Definitions

POAF was defined by the documentation of AF of any duration at any time in the postoperative period on a physician assessment, based on a rhythm strip or 12-lead electrocardiogram recording. On this basis, the patient population was divided into two arms according to the occurrence of POAF during CS and/or CR. An episode was counted as POAF only when AF was sustained for at least 30 s on a rhythm strip or 12-lead ECG; isolated atrial premature beats and shorter runs were not classified as POAF. During the surgical hospitalization, patients were monitored by continuous telemetry, whereas during in-hospital CR, rhythm was assessed by daily clinical evaluation, scheduled 12-lead ECG and telemetry/Holter when clinically indicated. Because monitoring intensity was not fully standardised across the 17 centres, residual under-detection of asymptomatic episodes—particularly during the CR phase—cannot be excluded and is acknowledged among the study limitations. Patients were further categorised according to whether POAF consisted of a single episode or of multiple (recurrent) in-hospital episodes (Table 1).

### 2.3. Study Endpoints and Follow-Up Data Collection

The primary endpoint was defined as the incidence of a composite outcome—all-cause mortality and/or non-fatal stroke—at 5-year follow-up in patients who underwent isolated CABG and were admitted to early in-hospital CR. Secondary endpoints included: (1) the prognostic impact of POAF duration and timing (e.g., ≥48 h); and (2) the association between AF recurrence during follow-up and the primary composite outcome. Follow-up data were collected through structured interviews using a standardised questionnaire administered by a physician or a trained nurse. In the event of patient death, information was obtained via telephone interviews with family members and/or verified by the patient’s general practitioner. AF recurrence during follow-up was defined as any AF episode documented after CR discharge on a 12-lead ECG, Holter monitoring or cardiac-device interrogation and reported by the patient, the treating cardiologist or the general practitioner; episodes were captured through the structured follow-up interview and review of available external documentation.

### 2.4. Sample Size Estimation

The sample size calculation was based on the study’s primary endpoint: the combined incidence of all-cause mortality and non-fatal stroke five years after discharge. Assumptions included a significance level (α) of 0.05, a power of 80% (β = 0.20), and an estimated POAF incidence of 30% after CABG. The expected event rate at 5 years was 24% in patients with POAF and 16.2% in those without. Based on these parameters, the required sample size was estimated at 960 patients (288 with POAF and 672 without). To account for potential dropouts, the total number of patients to be enrolled was estimated as 1382.

### 2.5. Statistical Analysis

All collected variables were analysed: continuous variables were summarised using mean, standard deviation, minimum, median, and maximum; categorical variables were presented as frequencies (*n*, %). Data were evaluated for the entire cohort and separately for patients with and without POAF occurring after CS and/or during in-hospital CR. Univariate analyses were conducted to identify POAF predictors and compare groups. Kaplan–Meier curves were used to estimate survival in patients with and without POAF. Survival distributions of patients with and without POAF were compared using the log-rank test. Stroke and death rates were calculated for both groups, followed by multivariate logistic regression to assess the independent prognostic value of POAF and other potential predictors of the primary outcome. Predefined secondary outcomes were analysed using univariate and/or multivariate methods as appropriate. Statistical significance was set at *p* < 0.05; hazard ratios with 95% confidence intervals were reported. The CHA2DS2-VASc score was modelled as ordinal categories (0–1 as reference, 2, 3 and ≥4) according to established clinical thresholds. The multivariable logistic model was built using a stepwise selection procedure, entering the variables associated with the outcome at univariate analysis with a *p*-value < 0.1. Multivariable analyses were performed on the 1015 patients with complete data for all model covariates. All analyses were performed using SAS software version 9.4 (SAS Institute Inc., Cary, NC, USA). by statisticians at the Clinical Trial Centre, IRCCS MultiMedica, Milan, Italy.

## 3. Results

### 3.1. Baseline Characteristics

Among the 1139 patients who underwent isolated CABG and early in-hospital CR, 391 (34.3%) experienced at least one episode of POAF during hospitalisation in the surgical or rehabilitation phases. Patients with POAF were significantly older and had a higher prevalence of hypertension, prior AF, and prior electrical cardioversion (all *p* < 0.05) (Table 1). The overall mean age was 67.7 ± 9.3 years; 80.9% were male. Enrolment reached 1139 of the planned 1382 patients (82.4%); observed event rates were lower than anticipated (15.4% vs. 10.0%, instead of the assumed 24% vs. 16.2%), and a post hoc calculation based on the observed proportions and group sizes (*n* = 391 vs. 748) indicated a statistical power of approximately 73% (α = 0.05, two-sided) to detect the univariate difference in the primary endpoint; accordingly, the loss of statistical significance of POAF in the multivariable model is best interpreted as consistent with confounding, while a type II error cannot be entirely excluded.

### 3.2. Primary Endpoint

#### Association Between POAF and the Primary Endpoint

**Figure 1:**
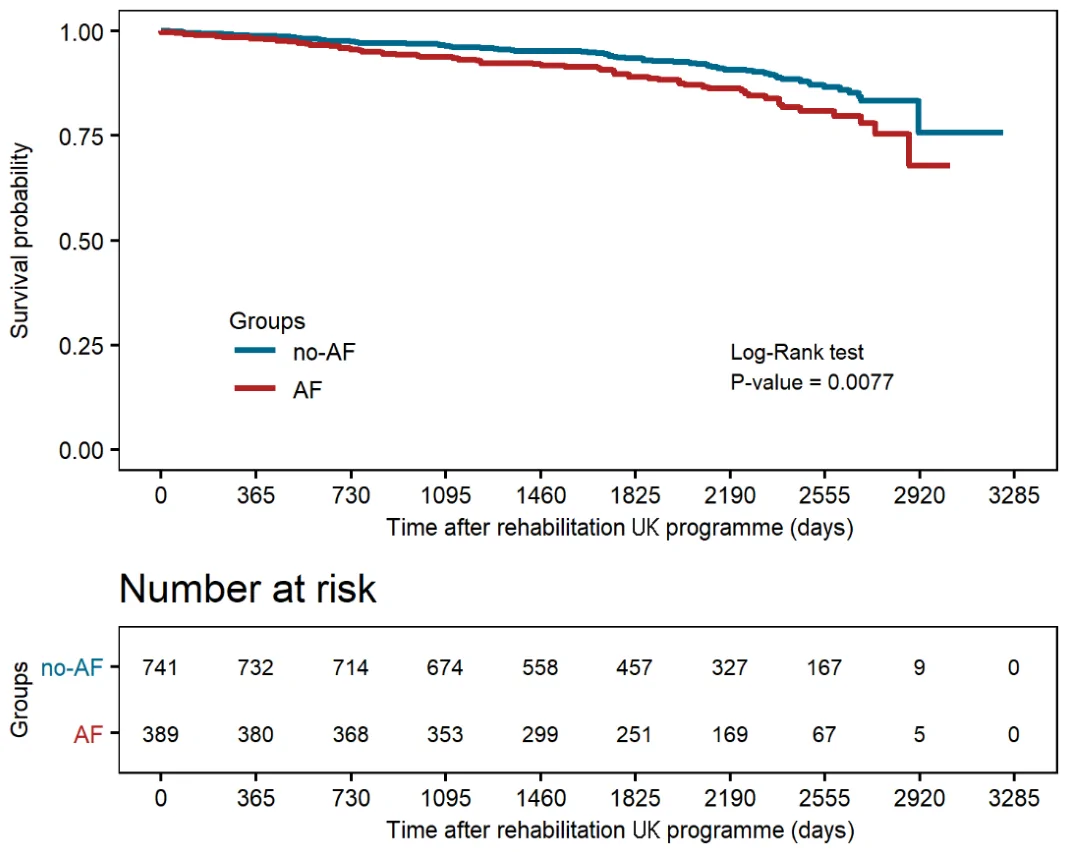
Probability of dead or non-fatal stroke occurrence during follow-up period according to presence of postoperative atrial fibrillation.

During a median follow-up of 5.78 years, the composite primary endpoint (all-cause death or non-fatal stroke) occurred in 135 patients (11.8%). This included 98 deaths (8.6%) and 46 non-fatal strokes (4.6%). The incidence of the primary endpoint was significantly higher in patients with POAF compared to those without (15.4% vs. 10.0%, *p* = 0.008) (Table 2). This association is further illustrated by Kaplan–Meier curves showing a higher cumulative event rate in patients with POAF (Figure 1). POAF lasting ≥48 h was strongly associated with the primary outcome (OR 8.3, *p* < 0.0001), while POAF <48 h showed a weaker association (OR 4.0).

**Figure 2:**
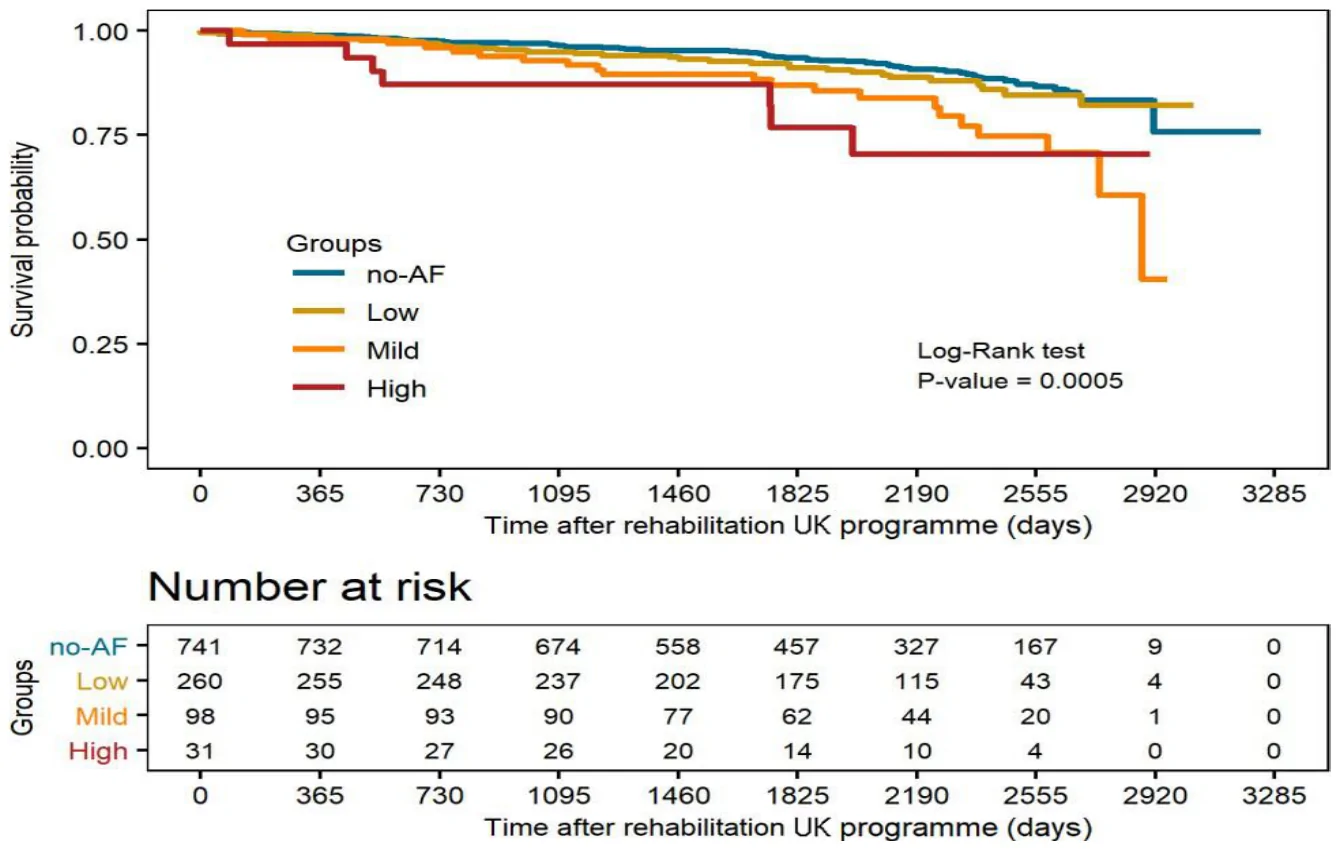
Primary endpoint during follow-up according to “POAF duration plus timing”. Risk categories are defined as follows: no POAF; low-risk POAF (occurring only during the surgical phase or only during rehabilitation and lasting <48 h); mild-risk POAF (occurring during both phases or lasting ≥48 h); and high-risk POAF (occurring during both the surgical and rehabilitation phases with a duration ≥ 48 h). Bars represent the incidence of the primary composite endpoint (all-cause death or non-fatal stroke) within each category.

Timing of POAF also influenced risk: the highest risk was observed in patients with POAF both during CS and CR (OR 10.3), followed by POAF only during CR (OR 5.4) and only during CS (OR 3.2) (Table 3). To better characterise risk among patients with POAF, we developed a composite classification based on the timing (during CS and/or CR) and duration (≥48 h or <48 h) of the arrhythmia. Four categories were defined: (1) no POAF; (2) low-risk POAF: occurring only during CS or only during CR and lasting less than 48 h; (3) mild-risk POAF: occurring either during both CS and CR or lasting ≥48 h; (4) high-risk POAF: occurring both during CS and CR with a duration ≥48 h. This classification showed a clear gradient in adverse event rates, with progressively higher incidence of the primary outcome across categories (Figure 2). Risk stratification based on POAF duration and timing also showed a graded increase in events, with the highest risk observed in patients with both prolonged and bi-phasic POAF (Figure 2). Antithrombotic and antiarrhythmic therapy at CABG discharge and during rehabilitation is summarised in Supplementary Table S1; at hospital discharge, oral anticoagulants were prescribed more frequently in patients with POAF (31.7% vs. 14.2%, *p* < 0.0001) and antiarrhythmic drugs far more frequently (61.6% vs. 3.8%, *p* < 0.0001), a pattern that persisted during rehabilitation.

### 3.3. Secondary Endpoints

#### 3.3.1. AF Recurrence and Association with Outcomes

AF recurrence after CR discharge occurred in 103 patients (10.1%). Its incidence was significantly higher in those with prior POAF (20%) compared to those without (5%) (*p* < 0.0001). AF during follow-up was significantly associated with the primary endpoint (14.6% vs. 5.7%, *p* = 0.0006), mainly driven by a higher incidence of non-fatal stroke (9.7% vs. 2.3%, *p* < 0.0001) (Table 4). Of the 103 patients with AF recurrence during follow-up, 69 (67%) had documented in-hospital POAF, in line with the higher recurrence rate observed in the POAF group (20% vs. 5%). The pattern of recurrent AF (paroxysmal vs. persistent) was not available.

#### 3.3.2. Independent Predictors of Mortality or Non-Fatal Stroke

At univariate analysis, the variables associated with the primary outcome at *p* < 0.1, and therefore entered into the stepwise multivariable model, were: age (*p* < 0.0001) and male sex (*p* = 0.002); family history of cardiovascular disease (*p* = 0.028), obesity (*p* = 0.091), hypertension (*p* = 0.059), diabetes (type 1, *p* = 0.046; type 2, *p* = 0.071), heart failure (*p* < 0.0001), peripheral arterial disease (*p* < 0.0001), chronic obstructive pulmonary disease (*p* = 0.002), chronic renal failure (*p* < 0.001), oncological disease (*p* = 0.054), previous stroke (*p* = 0.018), carotid artery disease (*p* < 0.0001), pacemaker carrier (*p* = 0.004) and previous peripheral revascularisation (*p* = 0.024); worse haemoglobin (*p* = 0.002), worse urea (*p* < 0.0001), worse creatinine (*p* < 0.001), worse glomerular filtration rate (*p* < 0.0001), worse glycaemia (*p* = 0.020) and worse uricaemia (*p* = 0.043); medical complications during cardiac surgery (*p* < 0.0001) and during rehabilitation (*p* = 0.027), LVEF during rehabilitation (*p* < 0.0001) and mitral regurgitation during rehabilitation (*p* = 0.075); a HAS-BLED category ≥ 3 (*p* = 0.014) and the CHA2DS2-VASc category (3, *p* < 0.001; ≥ 4, *p* < 0.0001); and, among treatments, antiarrhythmic drugs at rehabilitation discharge (*p* = 0.032) and, at the follow-up contact, anticoagulants (*p* = 0.022), antiarrhythmic drugs (*p* = 0.069), ezetimibe (*p* = 0.013) and n-3 PUFA (*p* = 0.059). Hypertriglyceridaemia (*p* = 0.101) and n-3 PUFA at discharge (*p* = 0.109) did not meet the entry threshold.

Multivariable analyses (Table 3, Table 4 and Table 5) and the AF-recurrence analysis were restricted to the 1015 patients (89.1%) with complete data for all model covariates; the 124 patients (10.9%) excluded from these models lacked one or more required variables (e.g., urea, LVEF, or follow-up rhythm data). A study flow diagram is provided (Figure S1), and baseline characteristics of included versus excluded patients are compared in Supplementary Table S2. Included and excluded patients were broadly comparable, the latter being slightly older and less frequently diabetic, with no other significant differences (Table S2). Multivariate logistic regression analysis identified several independent predictors of the primary composite endpoint (all-cause mortality or non-fatal stroke). A CHA_2_DS_2_-VASc score of 3 or higher was significantly associated with increased risk, with an odds ratio (OR) of 4.0 for a score of 3 and 5.8 for scores ≥ 4 (both *p* < 0.01). Left ventricular ejection fraction (LVEF) assessed during CR emerged as a protective factor, with a lower risk of events observed for each percentage point increase in LVEF (OR 0.95, *p* = 0.0009). Higher urea levels were also associated with a modest but significant increase in risk (OR 1.012 per mg/dL, *p* = 0.01). In addition, a history of heart failure (HF) (OR 3.9, *p* = 0.017) and the occurrence of AF during follow-up (OR 3.0, *p* = 0.0017) independently predicted adverse outcomes (Table 5).

Importantly, after adjusting for these factors, POAF, regardless of its duration or timing, was no longer an independent predictor of the primary endpoint but only its occurrence during follow-up. Because the cumulative incidence of the primary endpoint was relatively high (11.8%), the reported odds ratios should be interpreted as approximations that tend to overestimate the corresponding relative risks.

## 4. Discussion

POAF, defined as newly occurring AF in the early postoperative phase, is a frequent and clinically relevant complication, affecting approximately 30% to 50% of patients undergoing cardiac surgery [3,4,5]. In our cohort, POAF was associated with a higher incidence of the composite endpoint (all-cause mortality or non-fatal stroke) during univariate analysis. This aligns with previous studies indicating that POAF is linked to increased long-term risks. For instance, a meta-analysis reported a four-fold increased risk of stroke in patients with POAF compared to those without [6], and it is a risk factor for stroke, myocardial infarction, HF, and death [6,7,8,9,10,11]. However, in the current study, after adjusting for confounding factors, POAF did not remain an independent predictor in multivariate analysis, suggesting that while POAF reflects early postoperative vulnerability, its prognostic role is surpassed by other clinical and echocardiographic markers when considering long-term outcomes. POAF prognostic value may be mediated by other clinical variables, as was also described by Echahidi et al., demonstrating that POAF is associated with increased long-term mortality; however, they also noted that this association may be attenuated after multivariable adjustment, suggesting that POAF may act more as a marker of overall clinical vulnerability than as an independent prognostic factor [3]. Furthermore, several predictors of POAF (such as advanced age, ventricular dysfunction, and comorbidities) are themselves strongly associated with worse prognosis, suggesting a potential confounding role in the relationship between POAF and long-term outcomes [12]. Nevertheless, AF recurrence during follow-up was significantly associated with the primary endpoint, particularly driven by an increased incidence of non-fatal strokes. These findings provide novel insights, as this specific association was not demonstrated in a previous meta-analysis despite being investigated [13]. This emphasises the need for vigilant long-term monitoring of patients who develop POAF, as they are more susceptible to subsequent AF episodes and related complications. Our findings demonstrate that the combination of POAF duration and timing significantly influences long-term outcomes. Patients experiencing POAF both during the CS and CR phases, especially when lasting ≥48 h, exhibited the highest risk for adverse events. This is in line with current evidence, showing a relationship between POAF duration and decreased survival [14]. This stratification underscores the importance of monitoring POAF characteristics to identify patients at elevated risk. Multivariate analysis showed AF recurrence (as aforementioned), CHA_2_DS_2_-VASc score, worsening of urea levels, history of HF, and decline in LVEF as independent predictor factors of adverse outcomes. The CHA_2_DS_2_-VASc score emerged as a strong independent predictor of the composite endpoint in our study. Higher scores correlated with increased risks, reinforcing its utility in risk stratification post-CABG. This is consistent with the literature suggesting that the CHA_2_DS_2_-VASc score effectively predicts adverse outcomes in patients undergoing cardiac surgery [15]. In different settings, the existing literature indicates that impaired renal function—as reflected by increased blood urea nitrogen (BUN) or BUN/creatinine ratios—is associated with higher mortality in patients with AF and concurrent acute ischemic stroke [16]. Similarly, a decline in LVEF during follow-up has been linked to increased mortality among patients with HF with preserved ejection fraction [17]. Furthermore, a history of HF has been identified as a significant predictor of adverse outcomes in patients with AF [18]. However, these predictors have not been investigated in such patients, thus representing a novel finding.

The contrast between the strong univariate associations of POAF—including its duration and timing—and its loss of independent significance in the multivariable model deserves emphasis. Patients with POAF were significantly older and more frequently hypertensive, with higher CHA2DS2-VASc and HAS-BLED scores and a greater burden of in-hospital complications (Table 1); these same factors are themselves powerful determinants of mortality and stroke. The disappearance of an independent POAF effect after adjustment therefore indicates that the univariate association was largely mediated by this shared risk-factor burden, supporting the interpretation of POAF as a marker of global clinical vulnerability rather than a direct causal determinant of long-term events. Consistent with this, patients with POAF were significantly older (mean 70.0 vs. 66.4 years, *p* < 0.0001) and had higher CHA2DS2-VASc scores (mean 2.66 vs. 2.24, *p* < 0.0001) and more in-hospital complications than patients without POAF (Table 1), confirming a substantial overlap between POAF and the covariates that remained independently predictive.

Mechanistically, POAF probably reflects the combination of a pre-existing vulnerable atrial substrate (atrial fibrosis, dilation, advanced age) and transient perioperative triggers such as systemic inflammation, oxidative stress, catecholamine surge and fluid shifts. Once these transient triggers resolve, the short-lived arrhythmia per se may add little independent long-term risk, whereas the underlying substrate—captured by age, LVEF, renal function and the CHA2DS2-VASc score—continues to drive events and predisposes to AF recurrence. This framework is consistent with our finding that AF recurrence during follow-up, rather than the index postoperative episode, independently predicted adverse outcomes.

From a practical standpoint, the proposed four-tier classification combining POAF timing (surgical and/or rehabilitation phase) and duration (<48 h vs. ≥48 h) could be applied at CR discharge to identify patients—particularly those with prolonged and bi-phasic POAF—who may warrant more intensive and prolonged rhythm surveillance (e.g., scheduled ECG/Holter or wearable/implantable monitoring) and earlier reassessment of anticoagulation. Prospective validation will be required before such a scheme can be recommended for routine clinical use.

Whether POAF is a cause or a consequence of the underlying risk profile remains an open question, and our data favour the latter interpretation while not excluding a contributory causal role through atrial remodelling and subsequent recurrence. Several factors that may modulate this relationship—including a detailed lipid profile, glycated haemoglobin, hepatic function, retinal vascular assessment, and the prognostic impact of antithrombotic, antiarrhythmic and antidiabetic therapy—were not systematically collected in this retrospective cohort and could not be analysed; they represent relevant avenues for future prospective study. Renal function was retained in the model through urea, the variable consistently available across centres, whereas comprehensive hepatic-function data were not uniformly recorded.

This study has some limitations. Firstly, although observational in design, the enrolled cohort was retrospective, which may limit the generalisability of our conclusions. Secondly, monitoring intensity and POAF-detection methods were not fully standardised across the 17 participating centres, which may have produced differential under-ascertainment of asymptomatic episodes, particularly during the rehabilitation phase. Outcomes and AF recurrence were ascertained through structured interviews and general-practitioner verification rather than through centralised registries, introducing potential recall bias and under-detection of mild or silent events. The analytic cohort for the multivariable models was restricted to the 1015 patients with complete covariate data; although included and excluded patients appeared broadly comparable, residual selection bias cannot be excluded. Enrolment fell short of the pre-specified target, and observed event rates were lower than anticipated, yielding an estimated post hoc power of about 73% for the primary univariate comparison, so the non-significance of POAF in the multivariable analysis should be interpreted with the possibility of a type II error in mind. Data on lipid profile, glycated haemoglobin, and hepatic function during follow-up were not systematically available, precluding adjustment for these variables. Finally, because the primary-endpoint incidence exceeded 10%, the reported odds ratios should be read as approximations of relative risk.

## 5. Conclusions

In this multicentre study of patients undergoing isolated CABG and early CR, POAF was associated with worse outcomes at univariate analysis but did not independently predict long-term mortality or non-fatal stroke. Instead, AF recurrence during follow-up and a CHA_2_DS_2_-VASc score ≥ 3 emerged as strong independent predictors as well as a worse urea, a decrease in LVEF during CR and a history of HF in anamnesis. These findings highlight the importance of longitudinal rhythm monitoring and structured risk stratification after cardiac surgery; POAF duration and timing, with the highest risk observed in patients with both prolonged and bi-phasic POAF, can be a useful long-term risk marker. Further prospective studies are warranted to clarify whether targeted prevention or early intervention on AF recurrence may improve long-term outcomes. From a clinical standpoint, these findings argue for a risk-stratified rather than a one-size-fits-all approach to post-CABG management: instead of focusing exclusively on the transient postoperative arrhythmia, discharge planning should prioritise CHA2DS2-VASc-based risk stratification and structured long-term rhythm surveillance, with anticoagulation decisions guided by documented sustained or recurrent AF rather than by the isolated postoperative episode. Patients with prolonged and bi-phasic POAF may represent a particularly suitable target for intensified monitoring. These conclusions are aligned with the 2024 ESC/EACTS guidelines for the management of atrial fibrillation, which organise anticoagulation and follow-up around the individual thromboembolic risk profile within the AF-CARE pathway and recommend periodic reassessment; in the specific setting of postoperative AF after cardiac surgery, the guidelines highlight the increased long-term risk of stroke and AF recurrence and support long-term rhythm surveillance, with anticoagulation decisions guided by the individual risk profile rather than by the transient postoperative episode alone [19].

## Figures and Tables

**Table 1:** Characteristics of study population, stratified according to the occurrence of postoperative atrial fibrillation.

	No-POAF	POAF	Total	*Comparison* *No-AF vs. AF,* *p-Value*
N *(%)*	748 (65.67)	391 (34.33)	1139 (100.00)	*-*
Sex, *n* (%)				*0.301*
Female	136 (18.18)	81 (20.72)	217 (19.05)	
Male	612 (81.82)	310 (79.28)	922 (80.95)	
Age				*<0.0001*
Mean (SD)	66.44 (9.53)	70.01 (8.34)	67.67 (9.29)	
Median [IQR]	67 [61–73]	71 [65–76]	68 [62–75]	
Min–max	30–94	40–90	30–94	
Weight (Kg)				*0.408*
Mean (SD)	74.77 (13.25)	75.46 (13.30)	75.01 (13.27)	
Median [IQR]	73 [66–82]	74 [67–83]	74 [66–83]	
Min–max	43–170	40–134	40–170	
Height (cm)				*0.793*
Mean (SD)	167.03 (8.53)	167.17 (8.52)	167.08 (8.52)	
Median [IQR]	168 [162–172]	168 [161–173]	168 [162–172]	
Min–max	120–192	134–190	120–192	
Indication for CABG, *n* (%)				*0.214*
ACS	292 (39.73)	150 (39.58)	442 (39.68)	
Stable angina	273 (37.14)	126 (33.25)	399 (35.82)	
Silent ischaemia	88 (11.97)	45 (11.87)	133 (11.94)	
Other reason	82 (11.16)	58 (15.30)	140 (12.57)	
POAF occurrence, *n* (%)				*-*
Multiple	-	81 (29.24)	81 (29.24)	
Single	-	196 (70.76)	196 (70.76)	
Cardiovascular risk factor, *n* (%)				*0.699*
No	18 (2.41)	8 (2.05)	26 (2.28)	
Yes	730 (97.59)	383 (97.95)	1113 (97.72)	
LVEF (%)				*0.3771*
Mean (SD)	50.43 (9.99)	49.48 (11.29)	50.07 (10.50)	
Median [IQR]	52 [45–59]	50 [40–58]	51 [44–58.5]	
Min–max	20–75	20–75	20–75	
Coronary revascularisation, *n* (%)				*0.8250*
Incomplete	73 (11.18)	36 (10.71)	109 (11.02)	
Complete	580 (88.82)	300 (89.29)	880 (88.98)	
Cardiopulmonary bypass, *n* (%)				*0.0701*
No	231 (37.68)	146 (43.71)	377 (39.81)	
Yes	382 (62.32)	188 (56.29)	570 (60.19)	
Medical complication, *n* (%) (shown in Supplementary Table S1)				*0.0005*
No	431 (57.62)	183 (46.80)	614 (53.91)	
Yes	317 (42.38)	208 (53.20)	525 (46.09)	
Family history, *n* (%)				*0.536*
No	540 (72.19)	289 (73.91)	829 (72.78)	
Yes	208 (27.81)	102 (26.09)	310 (27.22)	
Obesity, *n* (%)				*0.054*
No	603 (80.61)	296 (75.7)	899 (78.93)	
Yes	145 (19.39)	95 (24.3)	240 (21.07)	
Hypertension, *n* (%)				*0.012*
No	206 (27.54)	81 (20.72)	287 (25.20)	
Yes	542 (72.46)	310 (79.28)	852 (74.80)	
Hypercholesterolaemia, *n* (%)				*0.565*
No	331 (44.25)	180 (46.04)	511 (44.86)	
Yes	417 (55.75)	211 (53.96)	628 (55.14)	
Hyperuricaemia, *n* (%)				*0.935*
No	738 (98.66)	386 (98.72)	1124 (98.68)	
Yes	10 (1.34)	5 (1.28)	15 (1.32)	
Hypertriglyceridaemia, *n* (%)				*0.764*
No	576 (77.01)	298 (76.21)	874 (76.73)	
Yes	172 (22.99)	93 (23.79)	265 (23.27)	
Diabetes, *n* (%)				*0.319*
No	474 (63.37)	236 (60.36)	710 (62.34)	
Yes	274 (36.63)	155 (39.64)	429 (37.66)	
Type 1	*17 (2.27)*	*9 (2.30)*	*26 (2.28)*	
Type 2	*257 (34.36)*	*146 (37.34)*	*403 (35.38)*	
Smoking habits, *n* (%)				*0.103*
No	387 (51.74)	228 (58.31)	615 (53.99)	
Past smoker	221 (29.55)	102 (26.09)	323 (28.36)	
Current smoker	140 (18.72)	61 (15.60)	201 (17.65)	
CHA_2_-DS_2_-VASc				*<0.0001*
Mean (SD)	2.24 (1.37)	2.66 (1.40)	2.38 (1.39)	
CHA_2_-DS_2_-VASc category, *n* (%)				*<0.0001*
0–1	241 (32.22)	86 (21.99)	327 (28.71)	*0.0003*
2	207 (27.67)	97 (24.81)	304 (26.69)	*0.2992*
3	159 (21.26)	93 (23.79)	252 (22.12)	*0.3290*
4+	141 (18.85)	115 (29.41)	256 (22.48)	*<0.0001*
HAS-BLED				*0.0033*
Mean (SD)	1.32 (0.90)	1.51 (0.97)	1.39 (0.93)	
HAS-BLED category, *n* (%)				*0.0326*
Low (0)	128 (17.11)	52 (13.30)	180 (15.80)	*0.0939*
Moderate (1–2)	543 (72.59)	281 (71.87)	824 (72.34)	*0.7946*
High (≥3)	77 (10.29)	58 (14.83)	135 (11.85)	*0.0244*

Abbreviations: ACS = acute coronary syndrome; CABG = coronary artery bypass graft surgery; IQR = interquartile range (1st quartile–3rd quartile); LVEF = left ventricular ejection fraction; POAF = postoperative atrial fibrillation; SD = standard deviation. Note. HAS-BLED categorisation was based on [2].

**Table 2:** Outcomes during follow-up period.

	No-AF (*n* = 748)	AF (*n* = 391)	Total (*n* = 1139)	Comparison No-AF vs. AF, *p*-Value
**Duration follow-up (years), median [IQR]**	5.74 [4.15–6.88]	5.81 [4.37–6.74]	5.78 [4.24–6.84]	*0.8814*
**Outcomes during follow-up, *n* (%)**				
**Primary—Dead or non-fatal stroke**	75 (10.03)	60 (15.35)	135 (11.85)	***0.0084***
** —Dead, *n* (%)**	52 (6.95)	46 (11.76)	98 (8.60)	*0.0060*
** —Non-fatal stroke**	26 (3.48)	20 (5.12)	46 (4.04)	*0.1821*
** —Fatal stroke**	1 (0.14)	4 (1.04)	5 (0.45)	*0.0504*

**Table 3:** Timing, duration of POAF and primary endpoint.

Independent Variables	Beta ± SE	*p*-Value	OR (95% CI)	N
POAF during CS or CR (ref. No)				
Duration < 48 h	1.390 ± 0.235	<0.0001	4.014 (2.532–6.363)	1015
Duration ≥ 48 h	2.112 ± 0.327	<0.0001	8.267 (4.356–15.690)	
POAF “where” (ref. No)				1015
Only during CABG hospitalisation	1.167 ± 0.256	<0.0001	3.212 (1.946–5.303)	
Only during rehabilitation	1.692 ± 0.418	<0.0001	5.431 (2.396–12.311)	
Both	2.337 ± 0.301	<0.0001	10.348 (5.734–18.674)	

CR: cardiac rehabilitation; CS: cardiac surgery; POAF: postoperative atrial fibrillation.

**Table 4:** Associations between AF occurrence during follow-up and outcome.

	No-AF Occurrence (*n* = 912)	AF-Occurrence (*n* = 103)	Total (*n* = 1015)	*p-Value*
Duration follow-up (years), median [IQR]	5.80 [4.25–6.87]	6.07 [4.73–6.85]	5.85 [4.28–6.86]	0.3615
Endpoints during follow-up, *n* (%)				
Primary—All-cause death or non-fatal stroke	52 (5.70)	15 (14.56)	67 (6.60)	0.0006
—All-cause death, *n* (%)	32 (3.51)	6 (5.83)	38 (3.74)	0.2404
—Non-fatal stroke	21 (2.3)	10 (9.71)	31 (3.05)	0.0000
—Fatal stroke	1 (0.11)	0 (0.00)	1 (0.10)	1.0000

**Table 5:** Multivariable logistic model. Dependent variable: primary outcome.

Independent Variable	Beta ± SE	*p*-Value	OR (95% CI)
CHA2-DS2-VASc category (ref. 0–1)			
2	0.178 ± 0.547	0.7450	1.195 (0.409–3.492)
3	1.397 ± 0.482	0.0037	4.045 (1.574–10.396)
4+	1.751 ± 0.458	0.0001	5.762 (2.346–14.151)
LVEF during CR (%)	−0.048 ± 0.015	0.0009	0.953 (0.926–0.981)
Worse urea	0.012 ± 0.005	0.0106	1.012 (1.003–1.021)
Heart failure in the history (ref. No)	1.368 ± 0.573	0.0168	3.929 (1.279–12.068)
Atrial fibrillation during follow-up (ref. No)	1.110 ± 0.354	0.0017	3.035 (1.517–6.074)

## Data Availability

The data underlying this study are not publicly available due to privacy restrictions but can be obtained from the corresponding author upon reasonable request and subject to approval by the participating institutions.

## Supplementary Materials

The following supporting information can be downloaded at: https://www.mdpi.com/article/10.3390/jcm15197577/s1, Figure S1. Study flow diagram. Figure S2. Forest plot of the univariate odds ratios (95% CI) for POAF duration and timing (Table 5). Table S1. Antithrombotic, antiarrhythmic and other pharmacological therapy at CABG discharge and during rehabilitation, by POAF status. Table S2. Baseline characteristics of patients included in versus excluded from the multivariable analysis.

## Abbreviations

The following abbreviations are used in this manuscript:

ACS	Acute Coronary Syndrome
AF	Atrial Fibrillation
BUN	Blood Urea Nitrogen
CABG	Coronary Artery Bypass Graft
CI	Confidence Interval
CR	Cardiac Rehabilitation
CS	Cardiac Surgery
ESC	European Society of Cardiology
HAS-BLED	Hypertension, Abnormal renal/liver function, Stroke, Bleeding, Labile INR, etc.
IQR	Interquartile Range
LVEF	Left Ventricular Ejection Fraction
OR	Odds Ratio
POAF	Postoperative Atrial Fibrillation
SAS	Statistical Analysis System
SD	Standard Deviation
