# Supplementary material for: Thromboembolic and Mortality Risk in Patients with Postoperative Atrial Fibrillation After Coronary Artery Bypass Graft—The TRIP-AF Study
Source: J Clin Med. 2026 Sep 29;15(19):7577. doi: 10.3390/jcm15197577 (PMC13666184; doi:10.3390/jcm15197577)
Supplement: Supplementary file 1 [file jcm-15-07577-s001.zip › jcm-4385792-supplementary.pdf]

Supplementary Materials

Thromboembolic and mortality risk in patients with postoperative atrial fibrillation after coronary artery bypass graft. The TRIP-AF Study.

Figure S1. Study flow diagram

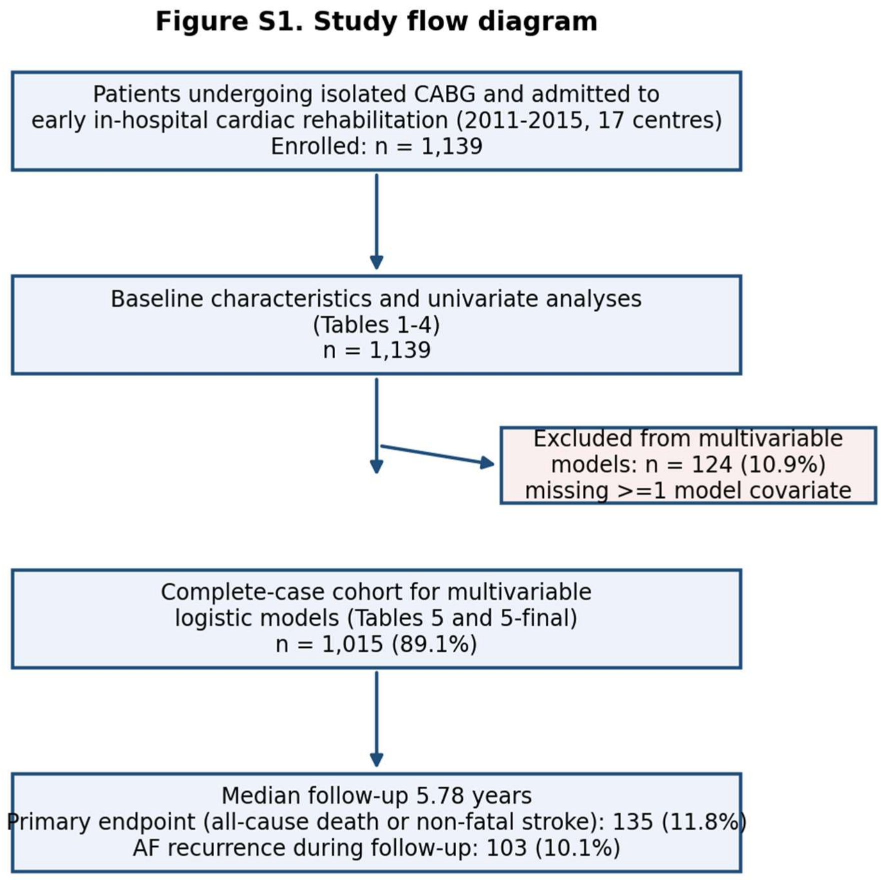

Figure S1. Flow of patients through the study. All enrolled patients contributed to baseline and univariate analyses; multivariable logistic models were restricted to patients with complete data for every model covariate.

Figure S2. Forest plot of POAF duration and timing (Table 5)

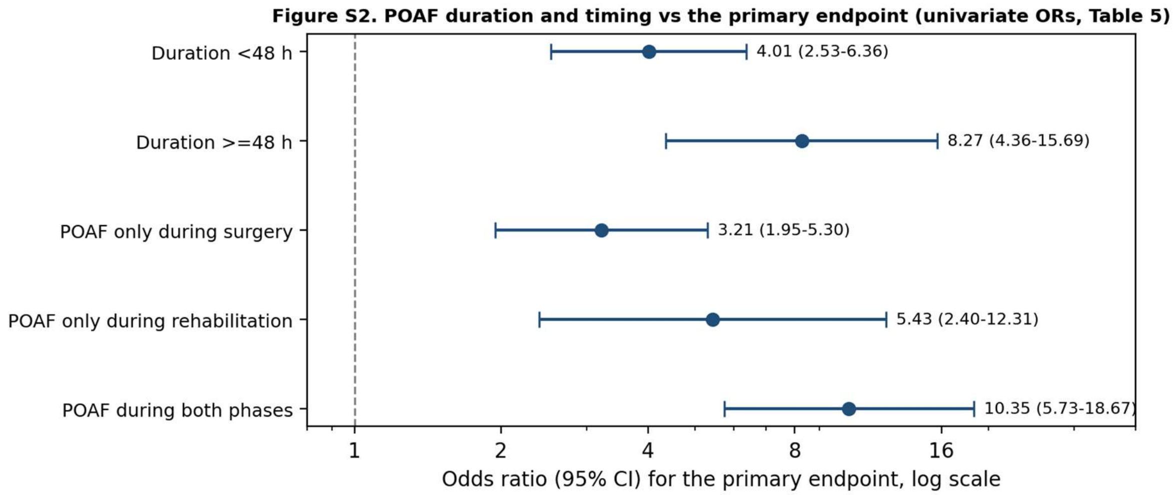

Figure S2. Univariate odds ratios (95% confidence intervals) for the primary composite endpoint according to POAF duration and timing, corresponding to Table 5. The dashed line indicates an odds ratio of 1.

Table S1. Antithrombotic, antiarrhythmic and other pharmacological therapy at CABG discharge and during rehabilitation, by POAF status

Therapy, n (%)	No-AF (n = 748)	AF (n = 391)	Total (n = 1,139)	p-value
At CABG hospital discharge				
**Renin-angiotensin system modulator**	336 (46.28)	196 (51.99)	532 (48.23)	0.0719
**Beta-blocker**	591 (80.52)	258 (67.01)	849 (75.87)	<0.0001
**Other heart rate modulator**	59 (8.22)	29 (7.77)	88 (8.07)	0.7990
**Antiplatelet**	737 (98.53)	377 (96.42)	1,114 (97.81)	0.0210
**Anticoagulant**	106 (14.17)	124 (31.71)	230 (20.19)	<0.0001
**Antiarrhythmic**	27 (3.76)	236 (61.62)	263 (23.87)	<0.0001
**Statin**	590 (80.59)	299 (77.47)	889 (79.52)	0.2161
**Ezetimibe**	6 (0.80)	11 (2.81)	17 (1.49)	0.0079
**n-3 PUFA**	94 (12.57)	30 (7.67)	124 (10.89)	0.0118
**Other therapy**	618 (84.21)	364 (89.64)	964 (86.15)	0.0142
During rehabilitation programme				
**Renin-angiotensin system modulator**	436 (60.72)	221 (58.01)	657 (59.78)	0.3817
**Beta-blocker**	664 (91.84)	318 (83.25)	982 (88.87)	<0.0001
**Other heart rate modulator**	82 (11.60)	35 (9.36)	117 (10.82)	0.2595
**Antiplatelet**	742 (99.20)	382 (97.70)	1,124 (98.68)	0.0350
**Anticoagulant**	85 (11.36)	101 (25.83)	186 (16.33)	<0.0001
**Antiarrhythmic**	12 (1.69)	163 (42.78)	175 (16.06)	<0.0001
**Statin**	652 (90.30)	331 (86.65)	983 (89.04)	0.0644
**Ezetimibe**	15 (2.01)	13 (3.32)	28 (2.46)	0.1721
**n-3 PUFA**	90 (12.03)	40 (10.23)	130 (11.41)	0.3638
**Other therapy**	492 (68.33)	299 (78.48)	791 (71.84)	0.0004

Table S1. Data are n (%). p-values compare patients with versus without POAF. AF, atrial fibrillation; PUFA, polyunsaturated fatty acids.

Table S2. Baseline characteristics of patients included in versus excluded from the multivariable analysis

Characteristic	Included (n = 1,015)	Excluded (n = 124)	p-value
**Age, years (mean ± SD)**	69.3 ± 8.6	71.1 ± 8.3	0.025
**Male sex, n (%)**	802 (79.0%)	94 (75.5%)	0.479
**POAF, n (%)**	313 (30.8%)	49 (39.2%)	0.063
**CHA2DS2-VASc, calculated (mean ± SD)**	2.9 ± 1.4	3.0 ± 1.3	0.424
**Hypertension, n (%)**	815 (80.3%)	95 (76.5%)	0.397
**Diabetes, n (%)**	443 (43.6%)	41 (33.3%)	0.031
**History of heart failure, n (%)**	27 (2.7%)	0 (0.0%)	0.064
**Prior stroke/TIA, n (%)**	112 (11.0%)	10 (7.8%)	0.392

Table S2. Comparison of patients included in versus excluded from the multivariable analysis (complete-case for POAF, CHA2DS2-VASc, LVEF and urea). Excluded patients were slightly older and less frequently diabetic; no other significant differences were observed, indicating essentially non-differential attrition. Percentages and means were derived from the eCRF and normalised to the official analysis denominators (1,015 included / 124 excluded; Tables 1-7); CHA2DS2-VASc was recomputed from age, sex and comorbidities.
